# Selenium-Enriched *Lactobacillus acidophilus* Ameliorates Dextran Sulfate Sodium-Induced Chronic Colitis in Mice by Regulating Inflammatory Cytokines and Intestinal Microbiota

**DOI:** 10.3389/fmed.2021.716816

**Published:** 2021-08-31

**Authors:** Zeyu Wu, Dan Pan, Min Jiang, Lixuan Sang, Bing Chang

**Affiliations:** ^1^Department of Gastroenterology, First Affiliated Hospital of China Medical University, Shenyang, China; ^2^Department of Geriatrics, First Affiliated Hospital of China Medical University, Shenyang, China; ^3^Department of Gastroenterology, Shengjing Hospital of China Medical University, Shenyang, China

**Keywords:** inflammatory bowel disease, ulcerative colitis, Se-enriched *Lactobacillus acidophilus*, intestinal flora, molecular pathological epidemiology

## Abstract

**Aim:** To evaluate the effect of Selenium-enriched *Lactobacillus acidophilus* (Se-enriched *L. acidophilus*) on dextran sulfate sodium (DSS)-induced colitis in mice.

**Methods:** Mice were randomly divided into four groups: a control group, a control + Se-enriched *L. acidophilus* group, a chronic colitis group, and a chronic colitis + Se-enriched *L. acidophilus* group (*n* = 10 each group). The mice were sacrificed on the 26th day. The disease activity index, survival rates, and histological injury score were determined. Cytokines produced by lamina propria lymphocytes (LPLs), the selenium (Se) concentrations in serum and colon tissue and the mouse intestinal microbiota were evaluated.

**Results:** Se-enriched *L. acidophilus* can improve histological injury and the disease activity index in mice with chronic colitis and reduce IL-1β, IL-6, IL-12p70, TNF-α, IL-23, IFN-γ, IL-17A, and IL-21 (*P* < 0.05) and increase IL-10 (*P* < 0.05) expression levels. Moreover, Se-enriched *L. acidophilus* can increase the β diversity of intestinal microbiota in mice with chronic colitis, significantly reduce the relative abundance of *Lactobacillus* and *Romboutsia* (*P* < 0.05), and significantly increase the relative abundance of *Parasutterella* (*P* < 0.05).

**Conclusions:** Se-enriched *L. acidophilus* can improve DSS-induced chronic colitis by regulating inflammatory cytokines and intestinal microbiota.

## Introduction

Inflammatory bowel disease (IBD) is a chronic recurrent inflammatory disease of the intestine that mainly includes two forms, ulcerative colitis (UC) and Crohn's disease (CD) (1), and its prevalence is increasing annually (2). The pathogenesis of IBD is not fully understood. Genes, immunity, intestinal flora, and the environment are all involved in the pathogenesis of IBD (3). There are a large number of microbiota in the human intestine, which has an important impact on the human body, and disorders of the intestinal flora are considered to be closely related to the occurrence and development of IBD (4). Studies have shown that the treatment methods for regulating the intestinal flora such as fecal bacteria transplantation (FMT) (5–7), and VSL#3 probiotic treatment (8, 9) could be used in the treatment of ulcerative colitis.

*Lactobacillus acidophilus* (*L. acidophilus*) is an important probiotic (10) that has a certain therapeutic effect on many diseases. *L. acidophilus* can alleviate the pain caused by osteoarthritis and delay the progression of osteoarthritis by reducing the destruction of cartilage and inhibiting the production of proinflammatory cytokines (11). It also has a certain relieving effect on type 2 diabetes (12). Obesity and fatty liver caused by diet can also be relieved by *L. acidophilus* through improving fat metabolism and insulin sensitivity (13). *L. acidophilus* can also inhibit endoplasmic reticulum stress (ER), thereby alleviating intestinal inflammation (14). In addition, evodiamine can relieve dextran sulfate sodium (DSS)-induced colitis by increasing L. acidophilus in the intestine (15).

Selenium (Se) is an important trace element in the human body that has antioxidant and anti-inflammatory effects and has an important influence on human immunity (16, 17). Clinical studies have found that compared with healthy people, CD patients exhibit significantly reduced concentrations of selenoprotein P and Se (18, 19), and the concentration of Se in UC patients is also significantly reduced (20). Moreover, it was reported that sodium selenite can alleviate DSS-induced colitis in mice (21).

The preparation of Se-enriched probiotics adopts the biological transformation method, in which inorganic Se is added during the probiotic culture process, and the probiotics take up inorganic Se and convert it into organic Se, which is then transformed into Se-enriched probiotics (22). Studies have found that Se-enriched probiotics can reduce liver damage induced by carbon tetrachloride (23, 24). The anti-inflammatory and antioxidant effects of Se-enriched probiotics can also improve the liver damage induced by heat stress in rats (25).

Since both Se and probiotics alleviate intestinal inflammation, Se-enriched probiotics may alleviate intestinal inflammation. Therefore, our study established a DSS-induced mouse colitis model to study the effect of Se-enriched *L. acidophilus* on intestinal inflammation and its possible mechanism.

## Experimental Method

### Experimental Animals and Probiotics

Forty 8-week-old specific pathogen-free C57BL/6 male mice were purchased from Liaoning Changsheng Biology, each weighing 22 ± 2 g and bred under specific pathogen-free conditions (temperature 21-25°C, humidity 50-60%, and a 12 h light/12 h dark-light regimen). Se-enriched *L. acidophilus* is a freeze-dried powder produced by the Immunology Laboratory of China Medical University. Each gram of freeze-dried powder contains Se-enriched *L. acidophilus* 5 × 10 ~ 9 cfu, and the selenium content is 0.30 mg/g. The research protocol was approved by the Animal Ethics Committee and Animal Care Committee of China Medical University. Ethics batch number: 2019069.

### Experimental Design

Forty mice were randomly divided into four groups: 10 in the control group (group A), 10 in the control + Se-enriched *L. acidophilus* group (group B), 10 in the chronic colitis group (group C), and 10 in the chronic colitis + Se-enriched *L. acidophilus* group (group D). The control group was given a normal diet and tap water, with normal saline gavage once a day. The control + Se-enriched *L. acidophilus* group was given a normal diet and tap water, with Se-enriched *L. acidophilus* (100 mg/kg) gavage once a day. The chronic colitis group was induced colitis by 1.5% DSS and given a normal diet with saline gavage once a day. The chronic colitis + Se-enriched *L. acidophilus* group was induced colitis by 1.5% DSS and given a normal diet with Se-enriched *L. acidophilus* (100 mg/kg) gavage once a day. Weight and disease activity index were recorded every day.

### Induction of Chronic DSS Colitis

Colitis was induced in the mice by oral administration of 1.5% DSS (molecular mass 36-50 kDa; MP Biomedicals, Solon, OH, United States) on days 0-5, 10-15, and 20-25 d and tap water on the other days (26). The mice were sacrificed on 26th day.

### Disease Activity Index

The disease activity index was used to assess the severity of colitis in mice. It consists of three parts, the percentage of weight loss (0-4 points), stool consistency (0-4 points), and intestinal bleeding (0-4 points) (26), as shown in Table 1. After the mice were sacrificed, the colon tissue was fixed with 4% paraformaldehyde, embedded in paraffin, cut into 4-μm sections, stained with hematoxylin and eosin, and scored for histological damage. Histological scores were assessed by two pathologists independently in a blinded fashion. The histological scores were obtained by calculating the sum of scores of inflammation severity, degree of mucosal damage, percentage of crypt damage, and pathological change range. The none, mild, moderate, or severe inflammation was quantified as to the percentage involvement by the inflammation (none, 0-33%, 33-67%, 67-100%). Depth of inflammation (none, mucous layer, submucosa, muscularis, and serosa) represented the mucosal damage, as shown in Table 2 (26).

**Table 1:** Disease activity index (DAI) score chart.

**Score**	**Weight loss (%)**	**Stool property**	**Bleeding**
0	0	Normal	Normal
1	>0-5		
2	>5-10	Loose	Fecal occult blood
3	>10-15		
4	>15	Diarrhea	Bleeding

**Table 2:** Histological injury score chart.

**Grade**	**0**	**1**	**2**	**3**	**4**
Inflammation	None	Mild	Moderate	Severe	-
Mucosal damage	None	Mucous layer	Submucosa	Muscularis and serosa	-
Crypt damage	None	1/3	2/3	100%	100% with epithelium loss
Pathological change range	None	0-25%	26-50%	51-75%	76-100%

### Cell Preparation, Culture, and Activation

The large intestine of each mouse was cut into 1- to 2-mm small pieces. The pieces were stirred twice in PBS containing 3 mmol/L EDTA for 15 min each and twice in RPMI 1640 (HyClone) containing 1 mmol/L EGTA for 20 min each to remove epithelium at 37°C. The remaining pieces were stirred in RPMI 1640 (HyClone) containing 20% fetal bovine serum, 100 U/ml collagenase (C2139; Sigma-Aldrich Corp., St. Louis, MO, United States) and 5 U/ml DNase1 (Sigma-Aldrich Corp) at 37°C for 90 min. The suspensions were centrifuged, and the particles were cleaned. Lamina propria lymphocytes (LPLs) were isolated from lamina propria (LP) cell preparations by 45-66.6% discontinuous Percoll (Solarbio) gradient centrifugation at 2,500 rpm for 20 min (26).

In an atmosphere containing 5% CO2, LPLs(1 × 10^5^/well in 0.2 ml of RPMI 1640 medium containing 10% fetal bovine serum, 1% penicillin, and 1% streptomycin) were cultured in 96-well plates coated with anti-CD3 (10 μg/ml e-Bioscience, San Diego, CA, United States) and soluble anti-CD28 (1 μg/ml, e-Bioscience) mAbs for 48 h at 37°C. After 48 h, the supernatants were collected, and the cytokine concentrations were determined by enzyme-linked immunosorbent assay (26).

### Enzyme-Linked Immunosorbent Assay

According to the manufacturer's instructions, cell culture supernatants were collected after centrifugation at 1,000 rpm for 10 min, and cytokine concentrations were measured using mouse immunoassay kits (R&D Systems Inc., Minneapolis, MN, United States). The levels of TNF-α, IL-1β, IL-6, IL-23, and IL-12p70 were measured in the supernatants without anti-CD3/anti-CD28 monoclonal antibody stimulation. The levels of IFN-γ, IL-17A, IL-22, IL-21, and IL-10 were measured in the supernatants with or without anti-CD28/anti-CD3 monoclonal antibody stimulation (26).

### Determination of Selenium in Serum and Colon Tissue

The selenium content in colon tissue was determined by fluorescence atomic absorption spectrometry. Serum selenium concentrations were detected in duplicate by inductively coupled plasma mass spectrometry (ICP-MS, Perkin-Elmer SCIEX ElAN 6000, US) (21).

### DNA Extraction and Amplification

The fecal samples of mice were transported to laboratory within 2 h with an ice pack. All samples were frozen immediately then and stored at −80°C. Realbio Genomics Institute (Shanghai, China) performed DNA extraction and amplification. The microbial DNA of the samples was extracted by a QIAamp FAST DNA Stool Mini Kit (Item No. 51604, Qiagen, Germany) according to the instructions. The integrity and concentration of total DNA were quality tested by a Thermo NanoDrop 2000 UV spectrophotometer and 1% agarose gel electrophoresis. Primers 341F 5′-CCTACGGGRSGCAGCAG-3′ and 806R 5′-GGACTACVVGGGTATCTAATC-3′ (with a specific barcode in the primer) were used to amplify the V3-V4 region of the bacterial 16 s ribosomal RNA gene by PCR(95°C for 3 min, followed by 30 cycles at 98°C for 20 s, 58°C for 15 s, and 72°C for 20 s and a final extension at 72°C for 5 min), and amplified fragments of approximately 500 bp were obtained.

### DNA Sequencing and Analysis

Realbio Genomics Institute (Shanghai,China) performed DNA sequencing and analysis. According to the manufacturer's instructions, the PCR products were extracted from 2% agarose gels and purified using the AxyPrep DNA Gel Extraction Kit (Axygen Biosciences, Union City, CA, U.S.). Amplicons were quantified using Qubit 2.0 (Invitrogen, U.S.). All quantified amplicons were pooled to equalize concentrations in order to sequence using Illumina HiSeq/MiSeq (Illumina, Inc., CA, USA) and PANDAseq (https://github.com/neufeld/ pandaseq, version 2.9) was used to overlap the paired end reads of 250bp on their 3 ends for concatenation into original longer tags.

OTUs were clustered according to 97% similarity using UPARSE (http://drive5.com/uparse/), and USEARCH (version 7.0.1090) was used to identify and remove chimeric sequences. Each representative sequence was annotated by RDP Classifier (http://rdp.cme.msu.edu/) based on RDP Database. The OTU profiling table and alpha diversity indices (including Chao1 index, Shannon index, Simpson index, observed species index, and PD-whole-tree diversity index) were achieved by the Python scripts of QIIME (version 1.9.1). Principal coordinate analysis (PCoA) based on weighted UniFrac distance and the Adonis test were implemented by R software (version 3.5.1). The microbiota differences between different groups were analyzed with linear discriminant analysis effect size (LEfSe) analysis software. The correlations between microbiota and cytokines were analyzed by R software (version 3.5.1).

### Data Analysis

The data are expressed as the mean ± standard error, and the Shapiro Wilk test was used for normality analysis. If the data conformed to a normal distribution and homogeneity of variance, analysis of variance or *t*-test was used. If the data conformed to a normal distribution and uneven variance, the Welch test or *t*' test was used. If the data did not conform to a normal distribution, a non-parametric test was used. *P* < 0.05 indicated that the difference was statistically significant. SPSS version 22.0 (SPSS, Inc., Chicago, IL, United States) was used for data analysis, and GraphPad Prism 6.0 (GraphPad Software, Inc., La Jolla, CA, United States) was used for drawing.

## Results

### Se-Enriched *L. acidophilus* Improves DSS Colitis

**Figure 1:**
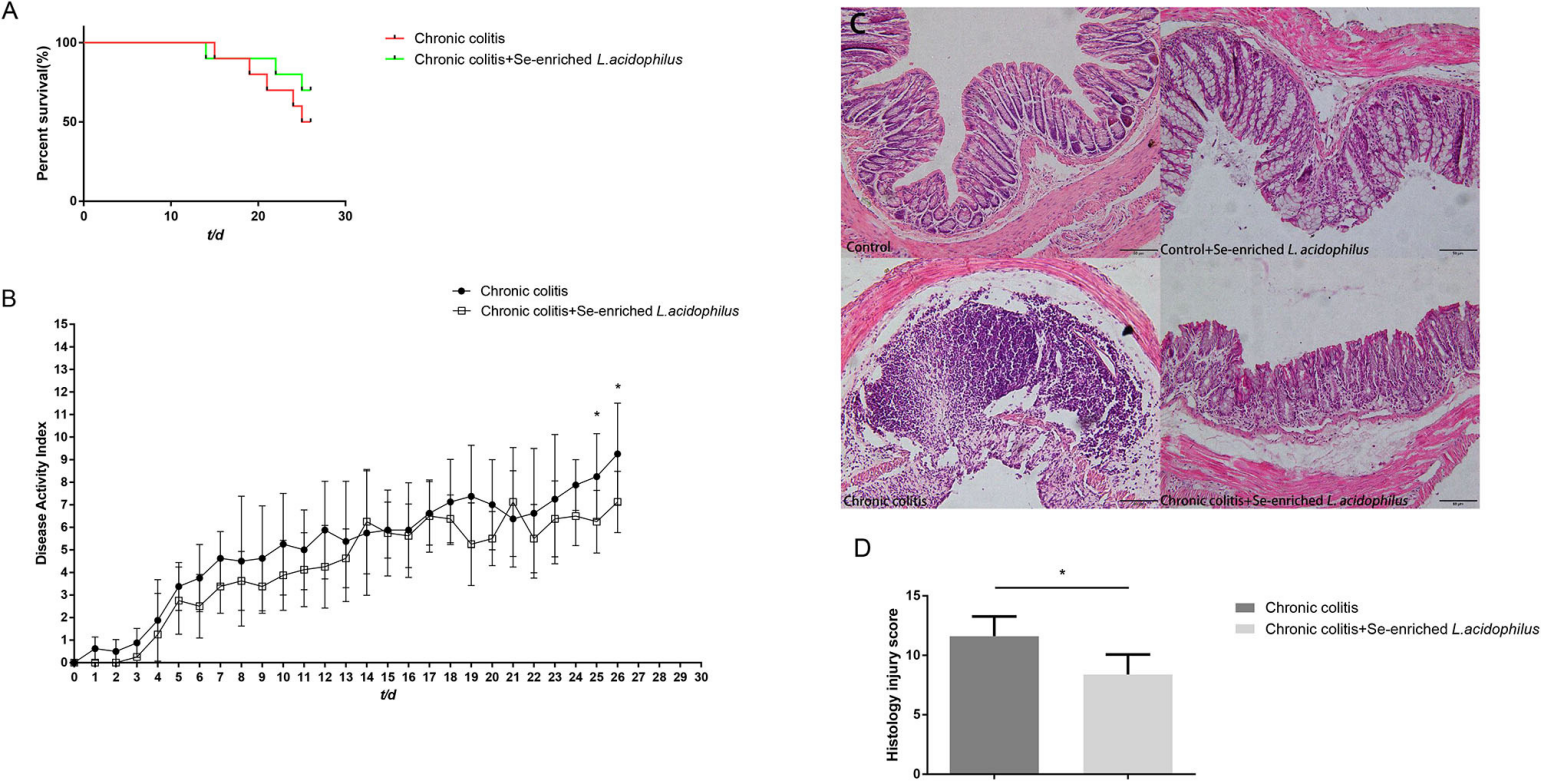
**(A)** Survival rates between the chronic colitis group and the chronic colitis + Se-enriched *L. acidophilus* group (*n* = 10); **(B)** Chronic colitis group and chronic colitis + Se-enriched *L. acidophilus* group DAI scores (*n* = 8); **(C)** H&E staining of colon tissue of four groups. (200×) (Control) There were no inflammatory cells infiltration. (Control+ Se-enriched *L. acidophilus*) There were no inflammatory cells infiltration. (Chronic colitis) Numerous neutrophil and mononuclear cells infiltration could be found. (Chronic colitis + Se-enriched *L. acidophilus*) There were fewer neutrophil and mononuclear cells infiltration than chronic colitis group. **(D)** Histological injury scores between the chronic colitis group and the chronic colitis + Se-enriched *L. acidophilus* group (*n* = 5). Data are expressed as the mean ± standard error (^*^P < 0.05).

The effect of Se-enriched *L. acidophilus* on DSS colitis was compared by the differences in survival rate, DAI score and colon histology between the two groups. The chronic colitis group and the chronic colitis + Se-enriched *L. acidophilus* group had similar survival rates (*P* > 0.05). The DAI score of the chronic colitis + Se-enriched *L. acidophilus* group decreased significantly on the 25th and 26th days (*P* < 0.05), and the histological injury score of the chronic colitis + Se-enriched *L. acidophilus* group significantly decreased (*P* < 0.05), as shown in Figure 1.

### Se-Enriched *L. acidophilus* Regulates Cytokines

**Figure 2:**
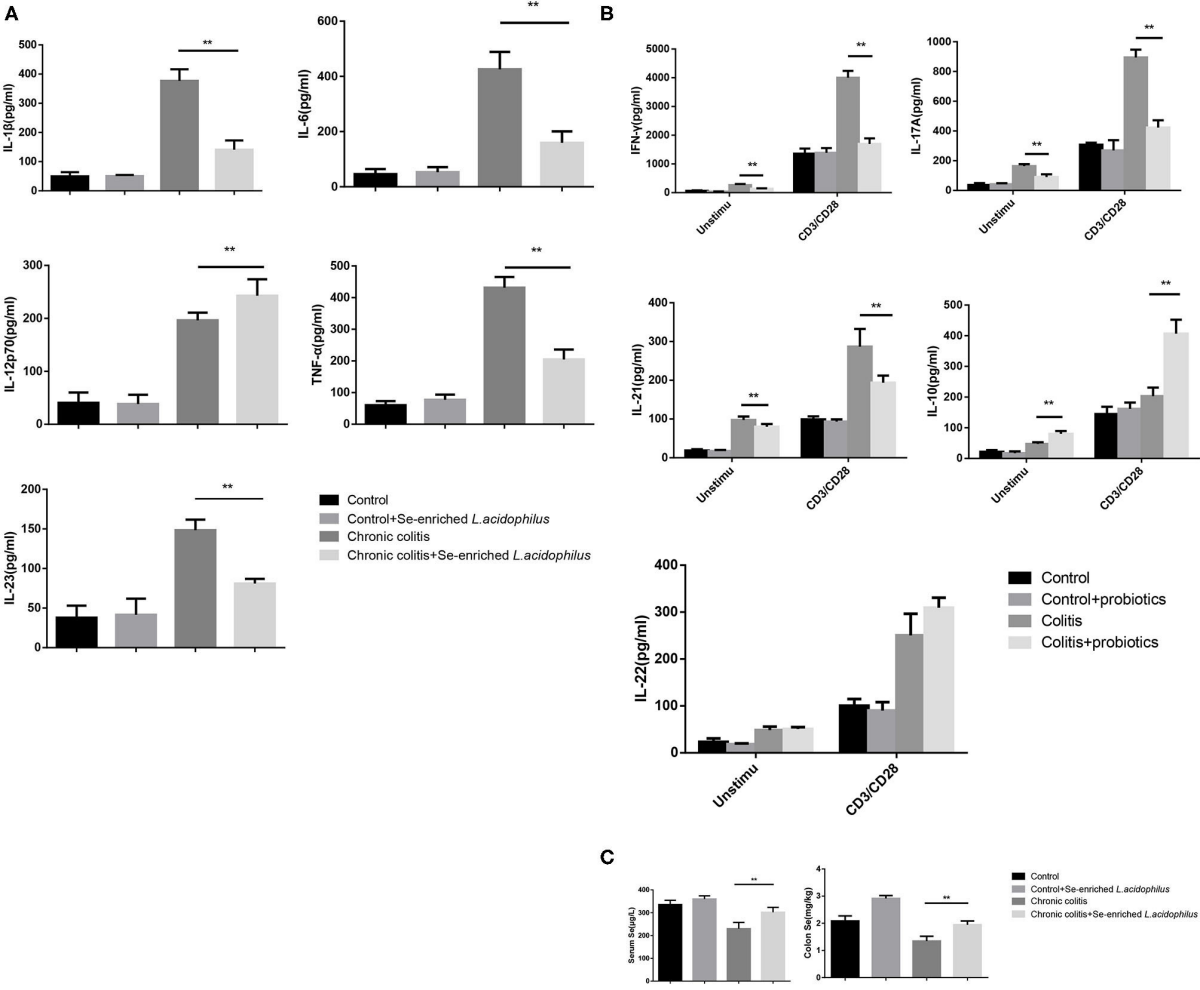
Cytokine concentrations produced by LPL cells and Se concentrations in serum and colon tissue. **(A)** Unstimulated cells; **(B)** LPL cells with or without anti-CD3 and anti-CD28 mAbs; **(C)** Se concentrations in serum and colon tissue. The values are expressed as the mean ± standard error. (**P < 0.01) (*n* = 5).

Detection of the cytokine concentrations in the supernatant (five samples per group) revealed that IL-1β, IL-6, IL-12p70, TNF-α, and IL-23 were significantly decreased in the chronic colitis + Se-enriched *L. acidophilus* group compared with the chronic colitis group (*P* < 0.05). Regardless of whether there was anti-CD3/CD28 antibody stimulation, IFN-γ, IL-17A, and IL-21 were significantly decreased (*P* < 0.05), and IL-10 was significantly increased in the chronic colitis + Se-enriched *L. acidophilus* group compared with the chronic colitis group (*P* < 0.05). Only the concentration of IL-22 between the two groups was not statistically significant. Compared with the chronic colitis group, serum and colon tissue Se concentrations were significantly higher in the chronic colitis + Se-enriched *L. acidophilus* group (*P* < 0.01), as shown in Figure 2.

### Se-Enriched *L. acidophilus* Regulates Intestinal Microbiota

**Figure 3:**
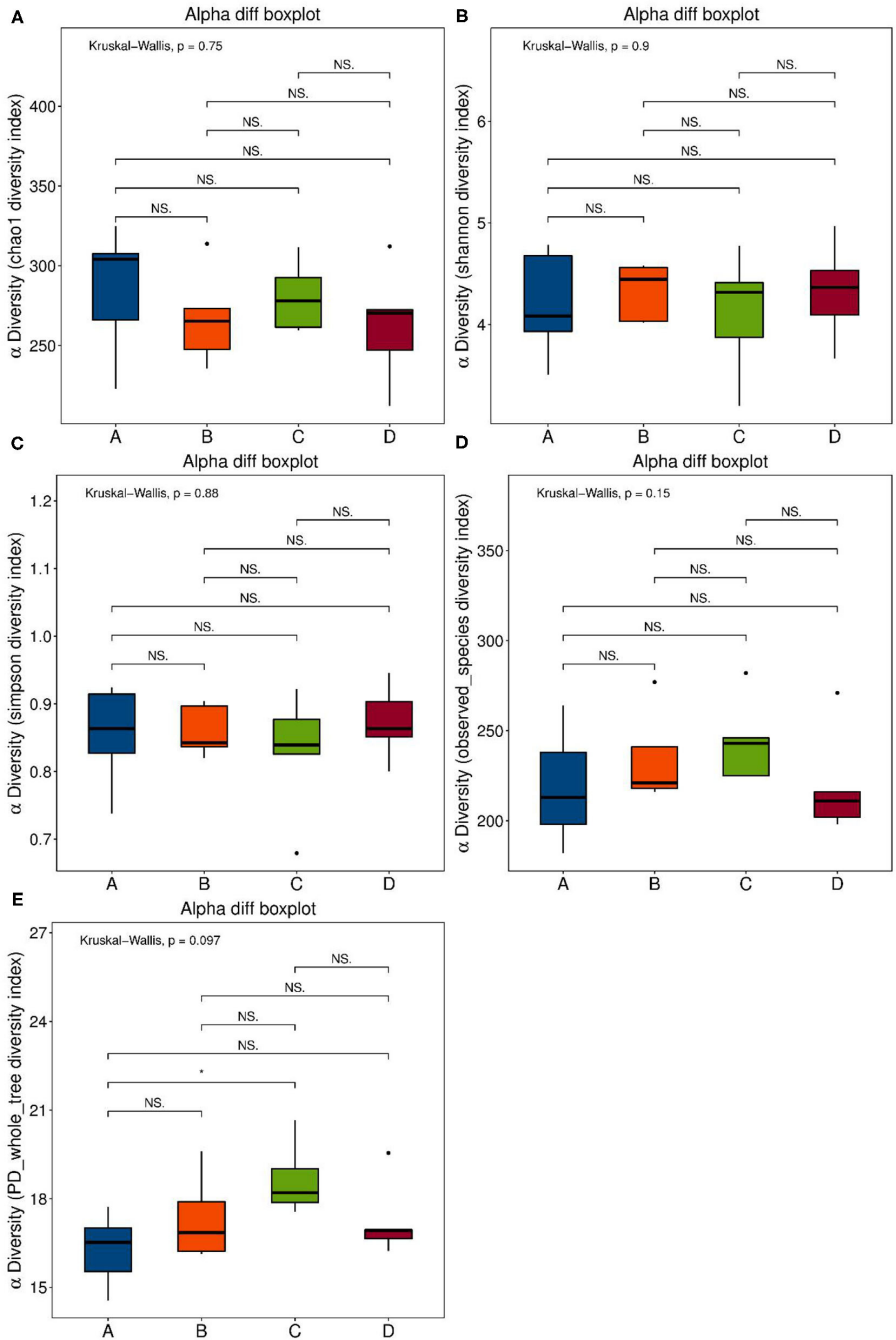
α diversity index. **(A)** Chao1 index: the differences between the four groups were not statistically significant; **(B)** Shannon index: the differences between four groups were not statistically significant; **(C)** Simpson index: the differences between four groups is not statistically significant; **(D)** Observed-species diversity: the differences between four groups were not statistically significant; **(E)** PD-whole-tree index: the differences between the four groups were not statistically significant.

**Figure 4:**
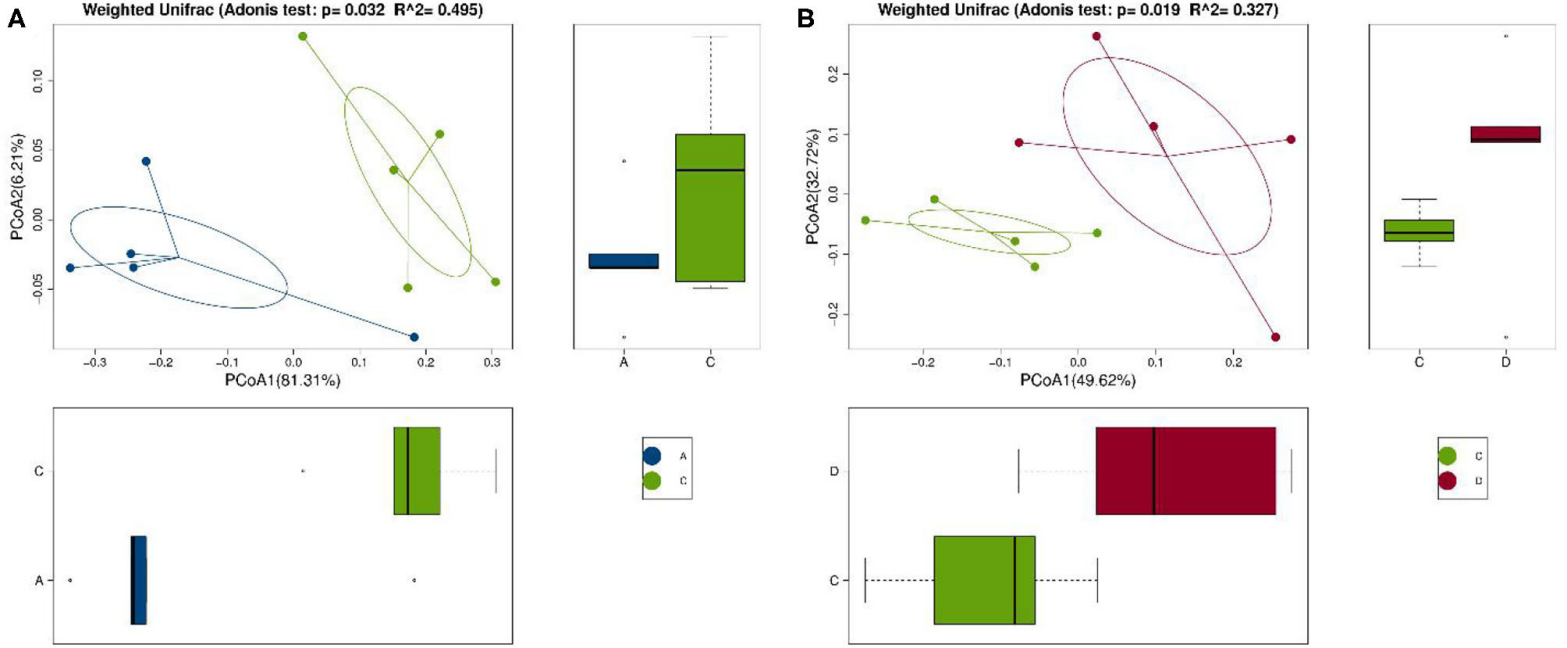
Comparison of β diversity among the control group (group A), the chronic colitis group (group C), and the chronic colitis + Se-enriched *L. acidophilus* group (group D). **(A)** The difference in β diversity between the control group (group A) and the chronic colitis group (group C) was statistically significant (*P* = 0.032 < 0.05); **(B)** The difference in β diversity between the chronic colitis group (group C) and the chronic colitis + Se-enriched *L. acidophilus* group (group D) was statistically significant (*P* = 0.019 < 0.05).

Se-enriched *L. acidophilus* has regulatory effects on the intestinal microbiota. The feces of mice in the control, control + Se-enriched *L. acidophilus*, chronic colitis, and chronic colitis + Se-enriched *L. acidophilus* groups were collected (five samples per group). There were no significant differences in the α diversity among the four groups, Chao1 index (*P* > 0.05), Shannon index (*P* > 0.05), Simpson index (*P* > 0.05), observed species index (*P* > 0.05), or PD_−_whole_−_tree diversity index (*P* > 0.05), as shown in Figure 3. There was a significant difference in microbial β diversity between the control group and the chronic colitis group (*P* < 0.05). There was also a significant difference in microbial β diversity between the chronic colitis group and the chronic colitis + Se-enriched *L. acidophilus* group (*P* < 0.05), as shown in Figure 4.

**Figure 5:**
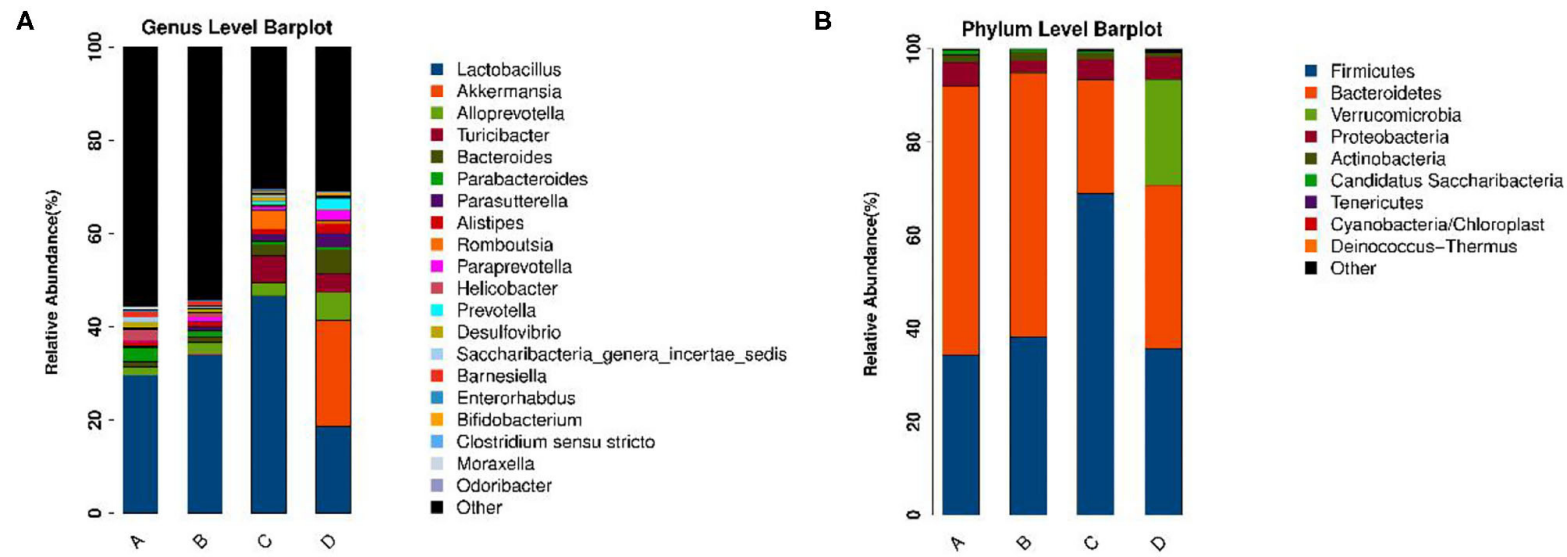
The relative abundance of each microorganism in each group. **(A)** Genus level; **(B)** Phylum level.

**Figure 6:**
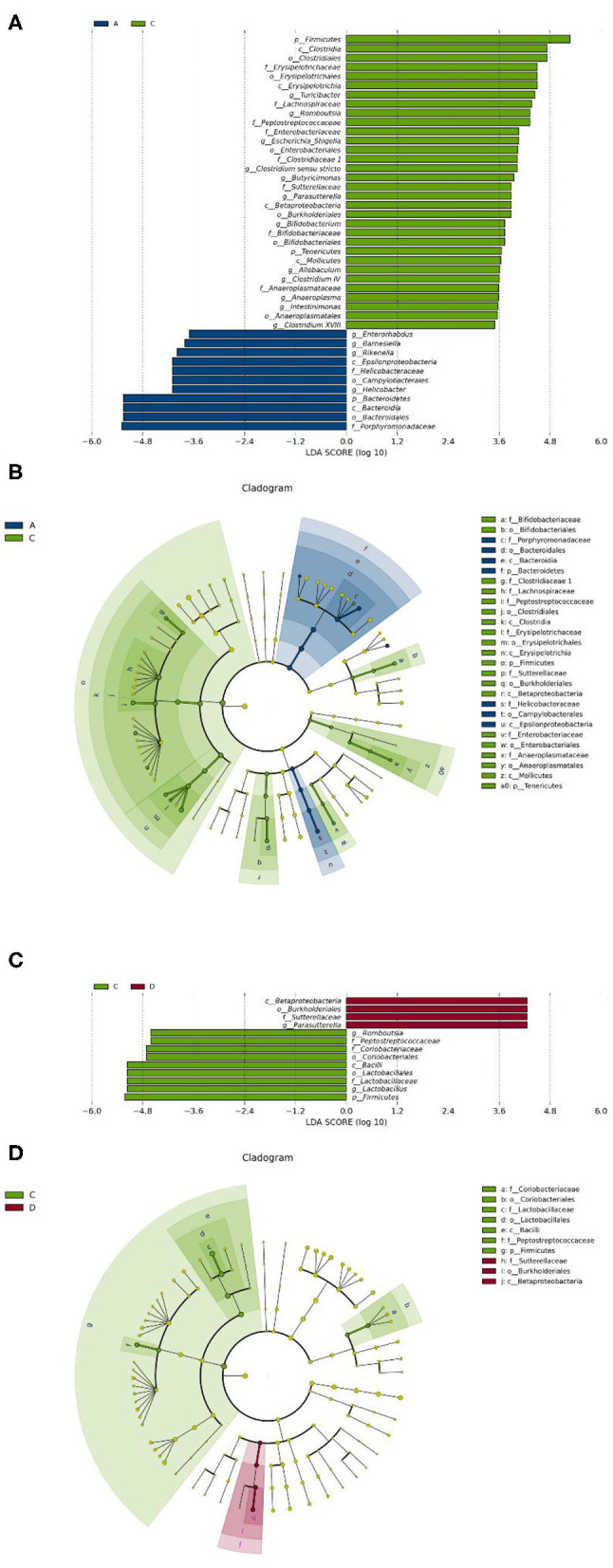
Se-enriched *L. acidophilus* changes the intestinal microbiota. **(A)** Marker bacteria (LDA score > 2) between the control group (group A) and the chronic colitis group (group C); **(B)** A LEfSe cladogram shows the dominant species in the control group (group A) and the chronic colitis group (group C); **(C)** Marker bacteria (LDA score > 2) between the chronic colitis group (group C) and the chronic colitis + Se-enriched *L. acidophilus* group (group D); **(D)** A LEfSe cladogram shows the dominant species of the chronic colitis group (group C) and the chronic colitis + Se-enriched *L. acidophilus* (group D).

**Figure 7:**
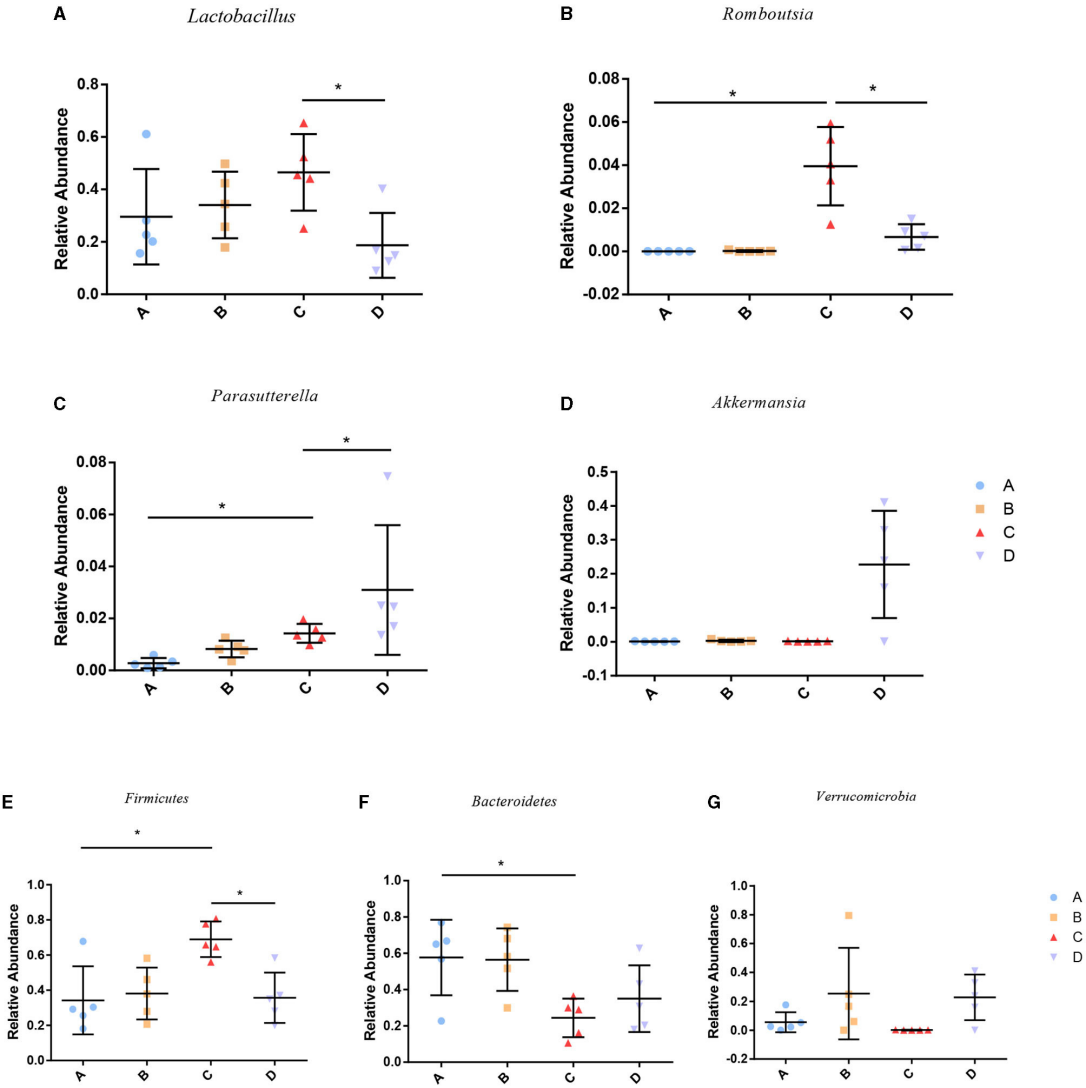
Relative abundance of species among groups. **(A)** *Lactobacillus*; **(B)** *Romboutsia*; **(C)** *Parasutterella*; **(D)** *Akkermansia*; **(E)** *Firmicutes*; **(F)** *Bacteroidetes*; **(G)** *Verrucomicrobia* (*P < 0.05).

To identify the differences in microbiota between different groups, we conducted LEfSe analysis of the dominant flora between different groups. There were differences in the composition of intestinal microbiota between the control group and the chronic colitis group and between the chronic colitis group and the chronic colitis + Se-enriched *L. acidophilus* group, as shown in Figure 5. At the phylum level, the control group was rich in *Bacteroidetes* (*P* < 0.05), and the chronic colitis group was rich in *Firmicutes* and *Tenericutes* (*P* < 0.05), as shown in Figure 6. At the genus level, compared with the chronic colitis group, the control group was rich in *Helicobacter, Rikenella, Barnesiella*, and *Enterorhabdus*, while *Turicibacter, Romboutsia, Escherichia_Shigella, Clostridium sensu stricto, Butyricimonas, Parasutterella, Bifidobacterium, Allobaculum, Clostridium IV, Anaeroplasma, Intestinimonas*, and *Clostridium XVIII* were significantly reduced (*P* < 0.05). The relative abundances of *Lactobacillus* and *Romboutsia* in the chronic colitis + Se-enriched *L. acidophilus* group significantly decreased (*P* < 0.05), and the relative abundance of *Parasutterella* significantly increased (*P* < 0.05), as shown in Figure 6. The relative abundance of *Akkermansia* increased, although the difference was not statistically significant [LDA score(log10) < 2], as shown in Figures 5, 7.

### Correlation Analysis Indicated That Many Species Were Correlated With Cytokines

**Figure 8:**
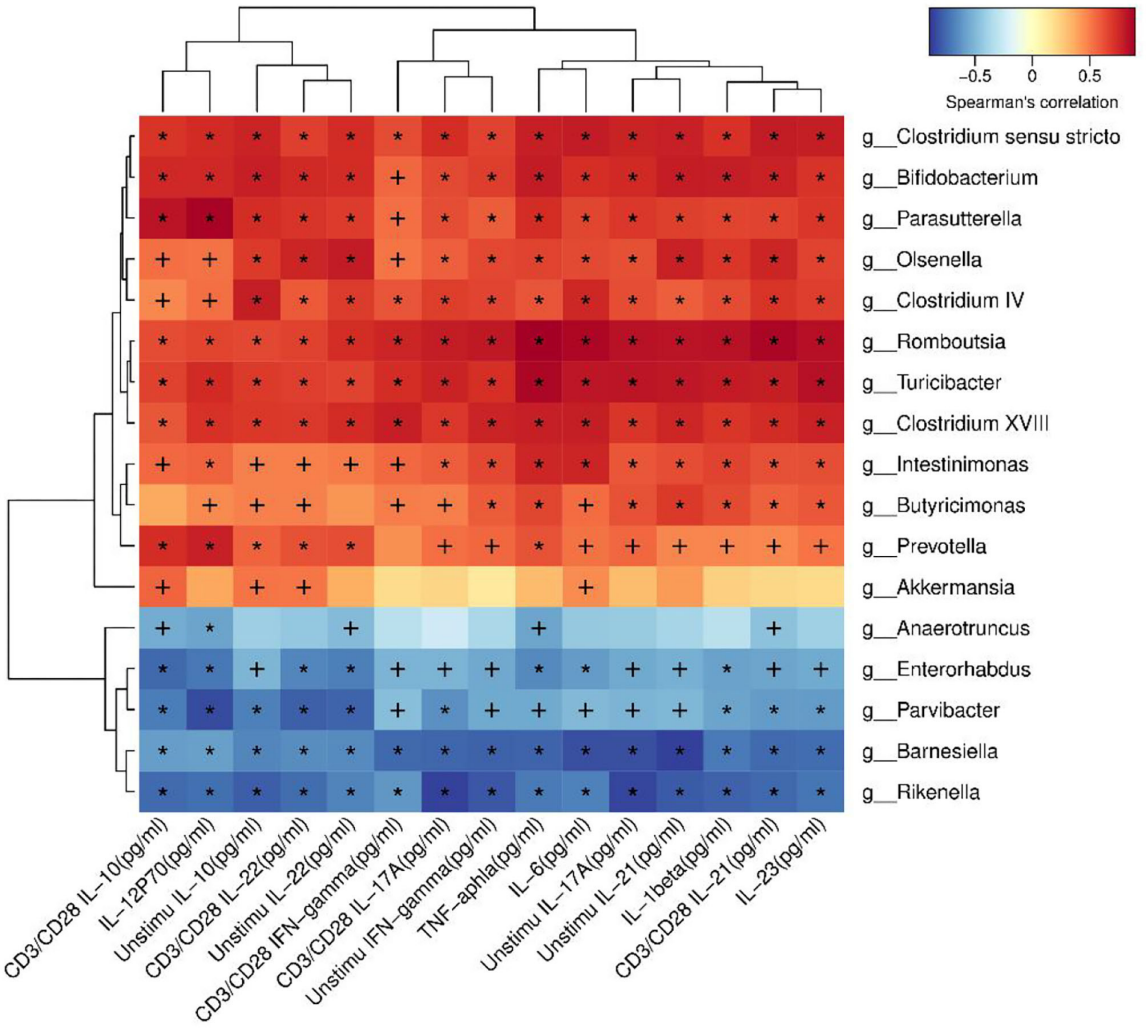
Correlation analysis between microbiota and cytokines.

In order to identify whether the alteration of microbiota were related to the cytokines, correlation analysis was performed. *Akkermansia* was positive related to IL-10 (*P* < 0.05). *Romboutsia* was positive related to TNF-α, IL-1β, IL-6, IL-23, IL-12p70, IFN-γ, IL-17A, IL-22, IL-21, and IL-10, as shown in Figure 8.

## Discussion

The beneficial effects of Se and *L. acidophilus* on IBD have been reported. Se-enriched *L. acidophilus* may have a certain therapeutic effect on IBD. We found that Se-enriched *L. acidophilus* could alleviate DSS-induced colitis in mice, reduce inflammatory cytokines produced by LPL cells, decrease the relative abundance of *Romboutsia* and *Lactobacillus*.

The serum Se concentration of IBD patients decreased (27). A Korean study showed that 30.9% of IBD patients had Se deficiency (28). Although the details of the relationship between Se and IBD still need further elucidation, animal studies have found that Se can increase CD4 (+) CD25 (+) regulatory T cells and reduce Th1, Th17, and γδ T cells, thus alleviating DSS-induced colitis (26). Se can transform M1 macrophages into M2 macrophages (29). M1 macrophages promote the development of inflammation, and M2 macrophages have anti-inflammatory effects (30).

*Lactobacillus* has long been considered a probiotic. Many studies have reported the therapeutic effects of different *Lactobacillus* strains on IBD. A randomized clinical trial showed that *Lactobacillus reuteri* ATCC 55730 enema combined with oral mesalazine can improve the intestinal inflammation of children with mild to moderate active distal ulcerative colitis (31). The *Lactobacillus rhamnosus* GG strain (LGG) plays a certain role in maintaining the remission stage of ulcerative colitis (32). Animal experiments showed that two *Lactobacillus reuteri* strains had therapeutic effects on colitis in mice (33). *Lactobacillus plantarum* 06CC2 has anti-inflammatory effects (34). At the same time, it was found that the intestinal *Lactobacillus* of mice with DSS-induced colitis decreased (35). This suggested that probiotics belonging to *Lactobacillus* may be benificial to colitis treatment. However, some studies also found the opposite result: *Lactobacillus* increased in IBD (36, 37), which is consistent with our result, which may suggest that the intestinal microbiota may have different changes in different stages of IBD. Our study found that Se-enriched *L. acidophilus* can reduce the relative abundance of *Lactobacillus*. A previous study also found that *Lactobacillus plantarum* could reduce the relative abundance of intestinal *Lactobacillus* in DSS-induced colitis mice (38). These indicate that although the changes in Lactobacillus in IBD need to be further clarified, some probiotics belonging to *Lactobacillus* may always have certain benefits for the treatment of IBD. Changes in the flora of IBD may be influenced by different situations (gene, diet, immunity, etc.) Therefore, molecular pathological epidemiology (MPE) may be useful for IBD research, and MPE can help doctors better understand the relationship between the flora and the disease. The changes in different flora may be used to distinguish different subtypes of IBD to facilitate more precise and effective treatments.

Se-enriched *L. acidophilus* can reduce the relative abundance of *Romboutsia*. It has been reported that the relative abundance of *Romboutsia* in the intestinal microbiota of patients with the autoimmune disease Hashimoto's thyroiditis is increased (39). Our study also found that the relative abundance of *Romboutsia* in DSS-induced colitis was increased, suggesting that *Romboutsia* may play a role in promoting autoimmune diseases, which needs further research.

Se-enriched *L. acidophilus* also increased the abundance of *Akkermansia* in mice with colitis, although there was no significant difference between the chronic colitis group and the chronic colitis + Se-enriched *L. acidophilus* group. Our study found that the relative abundance of *Akkermansia* was similar between the chronic colitis group and the control group. However, increase of the relative abundance of *Akkermansia* in the intestines of mice with DSS-induced colitis was also reported (40). *Akkermansia* plays an important role in the intestine. Chlorogenic acid and polyphenol-rich cranberry extract have been reported to alleviate colitis by increasing the abundance of *Akkermansia* (41, 42). *Akkermansia muciniphila*, a strain of *Akkermansia*, can maintain intestinal barrier function, reduce the inflammatory response, and alleviate DSS-induced colitis in mice (43). *Akkermansia muciniphila* extracelluar vesicles help to alleviate the progression of DSS-induced colitis (44). Se also has a certain effect on intestinal microflora. Zhai et al. reported that Se can increase the abundance of *Akkermansia* in the intestines of mice (45).

Se-enriched *L. acidophilus* also affects inflammatory cytokines. Cytokines play an important role in the pathogenesis of IBD. The expression levels of TNF-α, IL-1β, and IFN-γ in patients with IBD increased (46). *L. acidophilus* has inhibitory effects on the proinflammatory factors IL-6, IL-17, IL-1β, and TNF-α (47). Our study also found that Se-enriched *L. acidophilus* can inhibit the above proinflammatory cytokines. Another study found that *L. acidophilus* can improve endoplasmic reticulum stress and induce IL-10 production (14). IL-10 is an important anti-inflammatory cytokine in the human body (48). IL-10 can inhibit the production of IFN-γ by CD4+ T cells through dendritic cells (49). IFN-γ can increase intestinal vascular permeability and promote the development of intestinal inflammation (50). This mechanism may be related to *Akkermansia*. It has been reported that *Akkermansia* is positively correlated with IL-10 (43). We also found that *Akkermansia* is positively associated with IL-10. IL-21 can induce the initial T cells to differentiate into Th17 cells and produce IL-17 (51). IL-17 can promote the production of other inflammatory cytokines (52) and then promote the occurrence and development of inflammation. IL-23 can expand Th17 cells responses (53). IL-1β in synergy with IL-6 can promote the differentiation of Th17 cells (54). Therefore, inhibition of these inflammatory cytokines helps to alleviate inflammation.

There are still some limitations in this study. The specific mechanisms by which Se-enriched *L. acidophilus* regulates inflammatory cytokines are still unclear. The effect of Se-enriched *L. acidophilus* metabolites on colitis was not examined in these experiments. Further research should be conducted to clarify this series of problems.

In general, Se-enriched *L. acidophilus* can reduce the production of proinflammatory cytokines in DSS-induced colitis in mice, regulate the intestinal microbiota, and alleviate DSS-induced chronic colitis in mice. Therefore, Se-enriched *L. acidophilus* may have certain therapeutic effects on IBD, especially for patients with reduction of *Akkermansia* and IL-10, and clinical multicenter studies could be conducted to further study its efficacy in humans.

## Data Availability Statement

The original contributions presented in the study are included in the article/supplementary material, further inquiries can be directed to the corresponding author/s.

## Ethics Statement

All specimens from the mice were taken after ethical permission was obtained for participation in the study. The experimental protocols were approved by the Institutional Animal Care and Use Committee of China Medical University.

## Author Contributions

DP performed animal and molecular biology experiments. ZW and LS analyzed and interpreted the data and wrote the manuscript. MJ conceived and designed the study. All authors approved the final manuscript.

## Conflict of Interest

The authors declare that the research was conducted in the absence of any commercial or financial relationships that could be construed as a potential conflict of interest.

## Publisher's Note

All claims expressed in this article are solely those of the authors and do not necessarily represent those of their affiliated organizations, or those of the publisher, the editors and the reviewers. Any product that may be evaluated in this article, or claim that may be made by its manufacturer, is not guaranteed or endorsed by the publisher.
